# High population frequencies of *MICA* copy number variations originate from independent recombination events

**DOI:** 10.3389/fimmu.2023.1297589

**Published:** 2023-11-15

**Authors:** Anja Klussmeier, Kathrin Putke, Steffen Klasberg, Maja Kohler, Jürgen Sauter, Daniel Schefzyk, Gerhard Schöfl, Carolin Massalski, Gesine Schäfer, Alexander H. Schmidt, Axel Roers, Vinzenz Lange

**Affiliations:** ^1^DKMS Life Science Lab, Dresden, Germany; ^2^DKMS Group, Tübingen, Germany; ^3^Institute for Immunology, Medical Faculty Carl Gustav Carus, University of Technology (TU) Dresden, Dresden, Germany; ^4^Institute for Immunology, University Hospital Heidelberg, Heidelberg, Germany

**Keywords:** MICA, copy number variation, nonallelic homologous recombination, NAHR, linkage, population frequency, haplotype, *HLA-B27*

## Abstract

MICA is a stress-induced ligand of the NKG2D receptor that stimulates NK and T cell responses and was identified as a key determinant of anti-tumor immunity. The *MICA* gene is located inside the MHC complex and is in strong linkage disequilibrium with *HLA-B*. While an HLA-B*48-linked *MICA* deletion-haplotype was previously described in Asian populations, little is known about other *MICA* copy number variations. Here, we report the genotyping of more than two million individuals revealing high frequencies of *MICA* duplications (1%) and *MICA* deletions (0.4%). Their prevalence differs between ethnic groups and can rise to 2.8% (Croatia) and 9.2% (Mexico), respectively. Targeted sequencing of more than 70 samples indicates that these copy number variations originate from independent nonallelic homologous recombination events between segmental duplications upstream of *MICA* and *MICB*. Overall, our data warrant further investigation of disease associations and consideration of *MICA* copy number data in oncological study protocols.

## 1 Introduction

The *MICA* (MHC class I polypeptide-related sequence A) gene is located on chromosome 6 within the human major histocompatibility (MHC) complex, between *HLA-B* and *MICB* (1). Despite their high structural similarity to the classical human leukocyte antigen (HLA) genes, MICA and MICB (MICA/B) do not present peptides. Upon stress-induced expression by various cell types (e.g., epithelial cells, fibroblasts), MICA/B activate the NKG2D receptor, which is primarily found on NK cells and T cell subsets, thereby promoting immune cell recognition and immune surveillance (2–4).

Similar to the HLA genes, *MICA* is polymorphic with 531 currently known *MICA* alleles, encoding for 280 distinct proteins (IMGT-IPD/HLA database; release 3.53, 07/2023) (5). These *MICA* variants differ in the mechanism of cell surface attachment. While some are transmembrane proteins and can be shed from the membrane by proteolytic cleavage, the most frequent *MICA* allele, MICA*008, and other MICA*008-related alleles, are membrane-bound via a GPI-anchor and are recruited to exosomes (6–8). Nevertheless, both variants of soluble MICA (sMICA) can bind to NKG2D, which leads to receptor internalization. Decreased MICA cell surface expression and increased sMICA levels have been associated with inferior outcome in tumor patients and may represent an important cancer immune evasion principle (7–9). Consequently, innovative therapeutic approaches aim to increase the MICA/B density on the cellular surface by enhancing MICA/B expression and/or inhibition of MICA/B shedding (10–13). Some of these approaches might also be useful against infectious diseases since multiple viruses exploit similar strategies to avoid host cell recognition by immune cells (14–16).

Studies of *MICA* allele disease associations are hampered by the strong linkage disequilibrium of *MICA* and *HLA-B* (17). In transplantation, increasing evidence supports independent positive effects of *MICA* matching on outcome (18–21). In contrast, an independent role of *MICA* genotypes in addition to the well-established marker HLA-B*27 in ankylosing spondylitis (AS) remains questionable (22–24).

Gene copy number variations (CNV) contribute to human genomic diversity and arise from gene duplication or gene deletion events. Similar to single nuclear polymorphisms, some CNVs can be associated with disease phenotypes while others only seem to be benign polymorphic variations (25). *MICA* deletions have been identified in Asian populations with allele frequencies between 0.8% and 4% (26–28). The most common deletion is linked to HLA-B*48 and results from a 100 kb deletion between *HLA-B* and *MICB* (26, 29). So far, there are only controversial or very limited reports on disease associations, in particular nasopharyngeal carcinoma in Chinese patients (28, 30) and gout in Polynesians (31). To the best of our knowledge, *MICA* duplications have only been described as case reports, partly linked to leukemia (28, 32, 33).

Here, we present population frequencies of *MICA* copy number variations based on over two million individuals and a detailed characterization of the underlying genomic reorganization.

## 2 Methods

### 2.1 Samples

Volunteers from Germany (56%), UK (18%), Poland (15%), US (6%), Chile (4%), India (1%), and South Africa (0.4%) provided over two million samples to DKMS for registration as potential stem cell donors between May 2019 and October 2021. As part of the registration process, the donors are asked to self-assign to their ethnic background. Since selectable ethnicities vary between the different DKMS donor center questionnaires, data in this study are only grouped within one donor center (e.g., samples indicated as DE_Poland or PL_Poland share the Polish ethnic background, but one group registered with DKMS Germany and the other with DKMS Poland, respectively). The genotyping is within the scope of the consent forms signed at recruitment. For whole genome sequencing, pre-selected donors provided blood and written informed consent under a protocol approved by the Ethics Committee of the Technische Universität Dresden (EK 423092019). The study was conducted in accordance with the Declaration of Helsinki.

### 2.2 Genotyping

High-throughput genotyping is performed for *HLA-A*, *-B*, *-C*, *-E*, *-DPB1*, *-DQB1*, *-DRB1*, *-DPA1*, *-DQA1*, *-DRB3/4/5*, *MICA/B*, KIR, blood groups *ABO* and *Rh*, and *CCR5* as described before (34–39). In brief, DNA is isolated from buccal swabs and selected exons are amplified by PCR, followed by an additional barcoding PCR. After pooling and clean-up, the final sequencing libraries contain all amplicons of either 3480 or 7380 potential stem cell donors and are sequenced on a HiSeq 2500- (HiSeq Rapid SBS Kits V2 (500 cycles)) or NovaSeq 6000 (SP Reagent Kit (500 cycles)) instrument (Illumina, San Diego, USA), respectively. Data analysis is performed with neXtype and results are reported according to the current nomenclature (34, 40, 41). *MICA* and *MICB* are amplified in a multiplex PCR, in which the same primer pairs generate three amplicons for each gene. They cover exons 2 and 3 in separate amplicons and most bases of exons 4 and 5 in a third amplicon (35). Due to this restricted coverage, certain alleles cannot be resolved. These ambiguous genotyping results are abbreviated by using the most frequent allele of the allele group followed by a hash symbol (#): MICB*003/005/006/010 is reported as MICB*005#, MICB*004/028 is reported as MICB*004# (35). Since our sequencing data lack physical phasing, haplotype calls are based on frequency correlations from our population data (**Supplementary Data 1 and 2**). Consequently, rare haplotypes cannot be resolved.

Full-gene sequencing of *MICA* and *MICB* was conducted by long-range PCR with primer pairs located in the 5’- and 3’ UTR of the genes. Library preparation and sequencing was performed using SQK-LSK110 kits and MinION 9.4.1 flowcells following the manufacturer’s instructions (Oxford Nanopore Technologies, Oxford, UK). Sequences were analyzed with DR2S, an in-house developed software that is able to distinguish between alleles with varying gene copy numbers (42).

### 2.3 Identification of *MICA* copy number variations

For *MICA* genotyping, neXtypes algorithm first analyzes the sequencing reads of the three amplicons (exon 2, exon 3, exon 4/5) separately by calling exon allele groups (EAG) (34, 35). One EAG contains all known *MICA* alleles that share the same sequence in the given amplicon. These EAGs are then phased into *MICA* alleles under the assumption that only EAG combinations that can be found in the IPD/IMGT-HLA database are valid. For the classical HLA genes, up to two different EAGs per amplicon are allowed. For *MICA*, neXtype has calculated with up to three different EAGs per amplicon since May 2019. Consequently, it can detect three *MICA* gene copies in a sample (**Supplementary Table 1**, Sample B). Furthermore, in samples where only two different *MICA* alleles have been identified, a read coverage distribution of about 2:1 in all three amplicons also results in a genotyping result with three *MICA* gene copies (**Supplementary Table 1**, Sample C). Copy numbers of more than three can so far not be detected by neXtype.

In its current version, neXtype is only able to report hemizygous genotypes for KIR genes, but not for HLA or MIC genes. For this study, samples containing *MICA* deletions were identified retrospectively. For each sample, the number of mapped sequencing reads per exon was extracted from neXtype for both *MICA* and *MICB*. This number was used to calculate the ratio between *MICA*- and *MICB* sequencing reads for each exon, followed by the calculation of its mean and standard deviation (sd). All samples with a sequencing read coverage <100 in any exon or a sd >0.3, indicative of an uneven PCR amplification in one of the exons, were excluded. Additionally, samples with a heterozygous genotyping result in neXtype were excluded (n=21). In total, 2,089,638 samples (95%) passed all quality criteria and were used for further analysis (**Supplementary Figure 1**). For most samples, the calculated MICA/MICB ratio centered around 0.82 with only moderate variability. Two additional peaks were detected at ratios of 0.41 and 1.27 and represent MICA hemizygous (deletion) samples or samples with three MICA gene copies (duplications), respectively. All samples with a *MICA/MICB* ratio <0.53 were designated to be hemizygous for *MICA*.

### 2.4 Whole genome sequencing

One sample, pre-selected for harboring three different *MICA* alleles and HLA-B*27:02:01G, was subjected to whole genome sequencing. DNA was extracted from 4 ml blood using a chemagic™360 instrument according to the manufacturer’s instructions (PerkinElmer chemagen Technologie GmbH, Baesweiler, Germany). Small DNA was removed with Short Read Eliminator Kit XS (PacBio, Menlo Park, USA). PCR-free library preparation was performed with SQK-LSK112 kit (Oxford Nanopore Technologies, Oxford, UK) and the sequencing library was finally sequenced on MinION and PromethION flowcells with 10.3 chemistry to 20x sequencing depth (Oxford Nanopore Technologies, Oxford, UK). Assemblies were performed using shasta (43), Raven (44), miniasm/Racon (45, 46), or canu (47).

### 2.5 Targeted sequencing of recombination regions

PCR amplification and sequencing of the *MICA* duplication recombination region was performed using the primer pair MICA-Dup_M15: 5’-CAGTGCTGGATAGCATTTATGAGAC-3’and 5’-CTGCACAGTCACCCGCATGCAC-3’. 0.2 µM of each primer were mixed with genomic DNA (range: 6-300 ng), dNTPs (0.4 mM each), 1x Advantage Genomic LA Buffer and 1.25 U Advantage Genomic LA Polymerase Mix (Takara Bio, Mountain View, California) in a 25 µl reaction. PCR conditions: 94°C 1 min, 35 cycles: 98°C 10 sec/57°C 20 sec/68°C 5 min, 72°C 10 min. In the presence of a *MICA* duplication, a 4.9 kb fragment is amplified. Another 10 cycles of PCR were used to attach sample barcodes.

PCR amplification and sequencing of the *MICA* deletion recombination region was performed with primers DF (5’-AGAGTACAATCCATGTATAGAT-3’) and DR (5’-TTATCTCTTCTGTCCGTGAC-3’) according to Komatsu-Wakui et al. using the same reactions as described above (27). PCR conditions: 94°C 1 min, 35 cycles: 98°C 10 sec/55°C 20 sec/68°C 5 min, 72°C 10 min. In the presence of a *MICA* deletion, a 4.1 kb fragment is amplified. Another 10 cycles of PCR were used to attach custom sample barcodes. For some initial samples, primers DA and DS according to Komatsu-Wakui et al. were investigated (27). However, they could only be used for the HLA-B*48-linked *MICA* deletion due to primer mismatches in other haplotypes.

All PCR products were pooled and purified using 0.7x SPRIselect beads (Beckman Coulter, Brea, California). Library preparation was performed with SQK-LSK114 kits. Sequencing was performed on a GridION instrument and 10.4.1 flowcells according to the manufacturer’s instructions (Oxford Nanopore Technologies, Oxford, UK).

Sequencing reads were mapped against hypothesized references that were generated with the knowledge from our WGS sample and data from Komatsu-Wakui et al. (CLC Genomics Workbench 21 (Qiagen, Hilden, Germany)) (26, 27). These references contained the genomic region from *HLA-B* to *MICB*, using the CT dinucleotide repeat region around chr6:31389884 and chr6:31486863 as potential breakpoints (GRCh38/hg38). To exclude reads derived from unspecific amplification, the unmodified regions upstream of *MICA* and *MICB* were included in the reference. Only reads that exclusively mapped to the putative recombination region were finally used to calculate the breakpoint consensus sequence of each sample (**Supplementary Data 3**, GenBank accession numbers OR060976-OR061013).

### 2.6 Localization of the recombination site

The 20 kb upstream of *MICA* exon 1 and the 20 kb upstream of *MICB* exon 1 were extracted from publicly available genomic MHC assemblies to identify *MICA* and *MICB* specific bases in this highly homologous region. All sequences with larger assembly gaps or obvious assembly errors were excluded. Finally, 37 and 34 sequences for *MICA*- and *MICB* regions of diverse haplotypes were included, respectively (**Supplementary Data 4**) (48, 49). These sequences were aligned with the generated breakpoint consensus sequences of the *MICA* duplication- and *MICA* deletion samples (CLC Genomics Workbench 21 (Qiagen, Hilden, Germany)). In this alignment, a few bases could exclusively be assigned to either the region upstream of *MICA* or the region upstream of *MICB* in all available haplotypes (**Supplementary Data 5**). These marker SNPs were used to narrow down the presumed region of recombination for each sample.

## 3 Results

### 3.1 Detection of *MICA* copy number variations

We implemented amplicon-based genotyping for *MICA* and *MICB* as part of the genotyping profile for newly registered potential stem cell donors in 2017 (35). Frequently, the results were suggestive of three distinct *MICA* gene copies in one individual (**Supplementary Table 1**), which was confirmed by sequencing of the entire gene in selected samples (not shown). Consequently, we implemented the detection of three *MICA* gene copies into our NGS genotyping software neXtype (34).

Between May 2019 and October 2021, this workflow was used to process 2,188,836 samples. Among them were 22,880 samples (1%) with three copies of *MICA*. Apart from the *MICA* CNV, these samples had normal HLA genotyping results, including the *MICA* neighboring genes *HLA-B* and *MICB*.

Since neXtype cannot detect *MICA* deletions, we identified *MICA* hemizygous samples in this cohort retrospectively. In our workflow, the usage of identical primer pairs for *MICA* and *MICB* in a multiplex PCR reaction leads to a stable ratio of *MICA* to *MICB* sequencing reads. By using this *MICA*/*MICB* ratio, samples could clearly be separated according to their respective *MICA* gene copy number (**Supplementary Figure 1**). This identified 9,262 (0.44%) *MICA* hemizygous samples and confirmed our neXtype-based detection of MICA duplications with 97% concordance.

### 3.2 *MICA* duplications

#### 3.2.1 Linkage to HLA-B*27:02:01G

**Figure 1:**
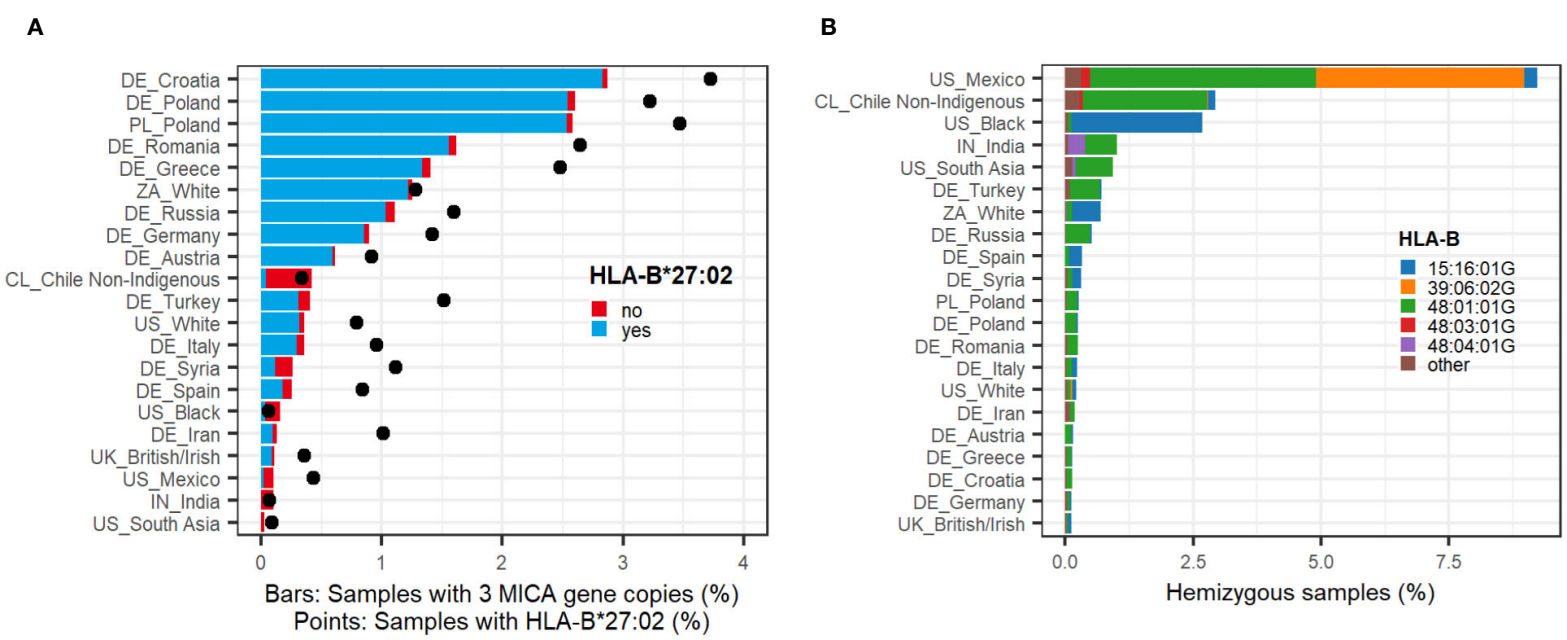
Population frequencies. **(A)** *MICA* duplications. Sample population frequencies of a *MICA* duplication in selected ethnic groups (n > 2500) (bars). Colors of the bars indicate whether samples have the common linkage to HLA-B*27:02:01G (blue) or any other haplotype (red). Black points depict the sample frequency of HLA-B*27:02:01G in the given population irrespective of *MICA*. All ethnic groups with n > 50 can be found in **Supplementary Data 6**. **(B)** *MICA* deletions. Sample population frequencies of *MICA* hemizygosity in selected ethnic groups (n > 2500). Colors of the bars indicate the linkage to frequently identified *HLA-B* alleles (**Table 1**). All ethnic groups with n > 50 can be found in **Supplementary Data 7**.

Of the 22,880 samples with a *MICA* duplication, 91% share one distinct haplotype: C*02:02:02G~B*27:02:01G~MICA*007~MICA*008~MICB*005# (40). A few of these samples were sequenced at higher resolution as C*02:02:02:03~B*27:02:01:01~MICA*007:01:01~MICA*008:01:02~MICB*005:02:03. Another 705 samples (3%) share the HLA-B*27:02:01G allele, but not the *HLA-C*- or *MICB* alleles. Overall, this indicates a strong linkage between HLA-B*27:02:01G and the *MICA* duplication. Indeed, in our dataset, the majority (68%) of HLA-B*27:02:01G-carriers possess the *MICA* duplication. However, the proportion of *MICA* duplication carriers among HLA-B*27:02:01G carriers varies with ethnicity. While around 83% of HLA-B*27:02:01G carriers harbor the *MICA* duplication in Poland or South Africa, only 13% of the HLA-B*27:02:01G positive individuals carry the duplication in Chile (**Figure 1A**).

Interestingly, we also identified novel *MICA* alleles that were exclusively found within the described HLA-B*27:02:01G-linked *MICA* duplication haplotype. The most frequent one (n=50) is a variation of the MICA*007 allele and was named MICA*243 (IPD/IMGT-HLA release 3.50; Accession number OX249873).

Even though the HLA-B*27:02:01G-linked haplotype is by far dominant, we identified *MICA* duplications in 1,260 samples without HLA-B*27:02:01G (5.5% of samples with a detected *MICA* duplication; 0.06% of total cohort). In these samples, we could not find another *HLA-B* allele that was predominantly linked to the *MICA* duplication other than the extremely rare alleles HLA-B*27:30 (n=6) and HLA-B*27:83 (n=3) (**Supplementary Data 1**). This suggests numerous independent recombination events.

#### 3.2.2 Population frequencies

Self-assigned ethnicities of the sample donors were used to calculate population frequencies of *MICA* duplications. Highest frequencies occur in the Eastern European populations with 2.9% in Croatia and 2.6% in Poland compared to 0.9% and 0.1-0.3% in Germany and Southern Europe/Great Britain, respectively (**Figure 1A**; **Supplementary Data 6**). In all studied European countries, the frequency of the *MICA* duplication clearly correlates with the population frequencies of HLA-B*27:02:01G, which indicates that it is predominantly driven by this one haplotype (**Figure 1A**). Nevertheless, *MICA* duplications are not completely absent in countries of non-European heritage and/or low HLA-B*27:02:01G allele frequency. For example, we identified *MICA* duplications in roughly 0.4% of the samples from Chileans with self-assigned non-indigenous heritage. In Mexicans, Indians, the American Black, and South Asian population, only few *MICA* duplications could be detected.

### 3.3 *MICA* deletions

#### 3.3.1 Haplotypes

In our dataset of 2,089,638 samples, 9,262 samples (0.44%) were identified as *MICA* hemizygous. In contrast to the *MICA* duplication, they cannot be associated with a single major haplotype. Instead, the *MICA* deletion is strongly linked to several *HLA-B* alleles, the majority to HLA-B*48:01:01G (n=5,747, 62%) (**Table 1**; **Supplementary Data 2**). Interestingly, this haplotype does not contain any functional MIC protein since it is also linked to the *MICB* null allele MICB*009N. However, other MICA deletion haplotypes, including HLA-B*48:04:01G, HLA-B*15:16:01G, and HLA-B*39:06:02:02 are linked to functional MICB alleles. The strong association of HLA-B*39:06:02:02 with a *MICA* deletion could only be determined after full gene sequencing. While only 2% of all HLA-B*39:06:02G carriers in our cohort have a *MICA* deletion, 77% of the samples with US Mexican ethnicity do. Sequencing of selected samples in full length revealed that HLA-B*39:06:02:01 is the dominant HLA-B*39:06:02G allele in Europe and is not linked to a *MICA* deletion. In contrast, HLA-B*39:06:02:02 was identified in all *MICA* deleted samples from the US Mexican population.

**Table 1:** Frequent *MICA* deletion haplotypes.

Linkage to *HLA-B*			*HLA-C~HLA-B~MICB* Haplotypes	
*HLA-B*	n (fraction of hemizygous samples)	Fraction hemizygous with *HLA-B*	Haplotype	n (fraction of hemizygous samples)
B*48:01:01G	5747 (62%)	96%	C*08:03:01G~B*48:01:01G~MICB*009N	2546 (27%)
			C*08:01:01G~B*48:01:01G~MICB*009N	2274 (25%)
			C*01:02:01G~B*48:01:01G~MICB*009N	264 (3%)
B*15:16:01G	1669 (18%)	94%	C*14:02:01G~B*15:16:01G~MICB*002	1316 (14%)
			C*14:02:01G~B*15:16:01G~MICB*005#	150 (2%)
			C*16:01:01G~B*15:16:01G~MICB*002	142 (2%)
			C*03:04:02G~B*15:16:01G~MICB*002	31 (0.2%)
B*39:06:02G	385 (4%)	2%*	C*07~B*39:06:02G~MICB*004#	373 (4%)
B*48:04:01G	137 (2%)	97%	C*01:02:01G~B*48:04:01G~ MICB*005#	87 (1%)
			C*08:01:01G~B*48:04:01G~ MICB*005#	48 (0.5%)
B*48:03:01G	122 (1%)	95%	C*04:01:01G~B*48:03:01G~MICB*009N	66 (0.7%)
			C*08:01:01G~B*48:03:01G~MICB*009N	50 (0.5%)
B*48:02:01	93 (1%)	100%	C*04:01:01G~B*48:02:01~MICB*009N	92 (1%)
B*48:39	52 (0.6%)	93%	C*08:01:01G~B*48:39~MICB*009N	51 (0.6%)
B*39:13:02	14 (0.2%)	100%	C*07~B*39:13:02~MICB*009N	13 (0.1%)
B*48:12	9 (0.1%)	77%	C*08:03:01G~B*48:12~MICB*009N	9 (0.1%)
**Other**	1034 (11%)			1750 (19%)
**Total**	9262 (100%)			9262 (100%)

Included are all HLA-B alleles and their most frequent haplotypes (HLA-C~HLA-B~MICB) that are at least 50% MICA hemizygous in a minimum of 10 genotyped samples. *HLA-B*39:06:02G is included because of 77% MICA hemizygosity in samples with Mexican ethnicity, which can be associated with HLA-B*39:06:02:02.

Nevertheless, and similar to the *MICA* duplications, not all *MICA* hemizygous samples could be assigned to a frequent haplotype (n=1,034, 11%). In these samples, a great variety of different *HLA-B* alleles was identified. Furthermore, only a few rare *HLA-B* alleles could be exclusively linked to *MICA* hemizygosity (**Supplementary Data 2**). For example, 96% of the samples with HLA-B*48:01:01G are *MICA* hemizygous, but 4% are *MICA* heterozygous.

#### 3.3.2 Population frequencies

The highest population frequencies of *MICA* hemizygosity were detected in people with Mexican or South American heritage. In Mexicans, who registered with DKMS in the US, a *MICA* deletion was identified in over 9% of the samples (**Figure 1B**; **Supplementary Data 7**). Among them are two major haplotypes, linked to either HLA-B*48:01:01G or HLA-B*39:06:02G. In Chileans, the HLA-B*48:01:01G haplotype dominates, while the US Black population is almost exclusively linked to HLA-B*15:16:01G, both with frequencies over 2.5%. In Indians and the US South Asian population, a linkage to HLA-B*48:04:01G could additionally be identified. In sharp contrast to these populations, *MICA* hemizygosity is consistently less frequent in European populations (around 0.3%).

### 3.4 Recombination

**Figure 2:**
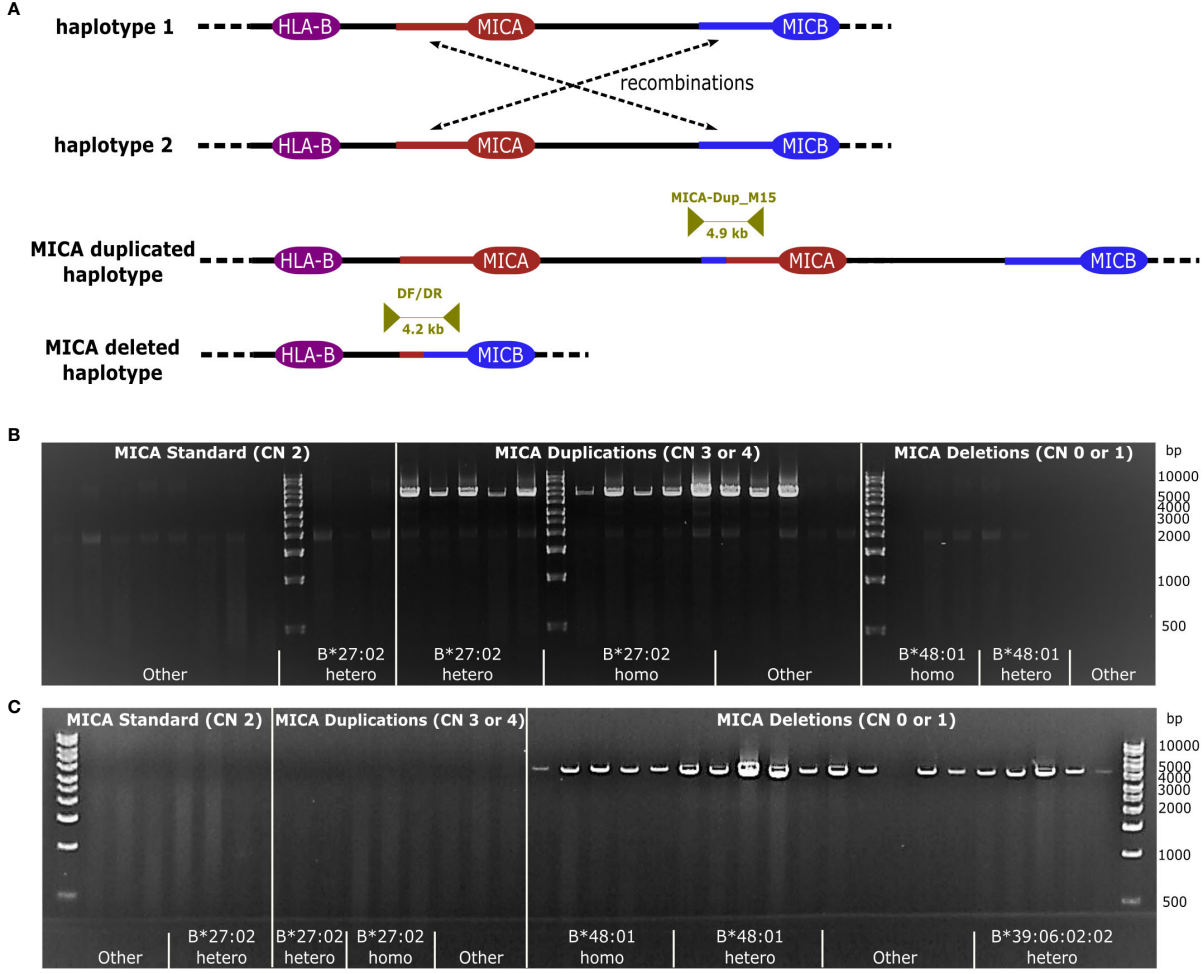
Recombination by NAHR. **(A)** Schematic representation of NAHR. Recombination takes place between two segmental duplications upstream of *MICA* and *MICB*, respectively (red/blue line). Either a *MICA* duplicated- or a *MICA* deleted haplotype can be generated. Dark yellow arrows indicate the primer pairs MICA-Dup_M15, specific for the *MICA* duplication, and DF/DR, specific for the *MICA* deletion, enclosing the anticipated recombination site. **(B, C)** Primer specificity. Samples with a *MICA* copy number (*MICA* CN) between 0 and 4 were subjected to a PCR with MICA-Dup_M15 **(B)** or DF/DR **(C)**. Samples included homo- and heterozygous samples of the dominant haplotypes linked to HLA-B*27:02:01G and HLA-B*48:01:01G as well as randomly chosen haplotypes (designated as Other) as well as HLA-B*27:02:01G genotyped samples without a *MICA* duplication. PCR failures could be caused by variations in the primer binding sites due to unavailable intergenic sequence information for many MHC haplotypes.

We performed long-read whole genome sequencing of a sample with three distinct *MICA* alleles and HLA-B*27:02:01G to examine the molecular mechanisms underlying our observations. Despite recovering all three *MICA* alleles in the whole genome data, assemblies with a variety of algorithms failed to reveal the genomic location of the duplication. We hypothesized that the 30 kB long segmental duplication upstream of *MICA* and upstream of *MICB* (93% sequence identity) might drive the duplication events by nonallelic homologous recombination (NAHR) (**Figure 2A**) (50–52). Manual inspection of the read mapping in this region indicated a potential breakpoint. To confirm this hypothesis, we sequenced a 4.9 kB PCR product spanning the predicted recombination region. In the case of a MICA duplication, a NAHR event should result in an amplicon that starts with a sequence otherwise located upstream of MICB and end with a sequence otherwise located upstream of MICA. Indeed, the obtained sequence was in full agreement with such an event.

**Figure 3:**
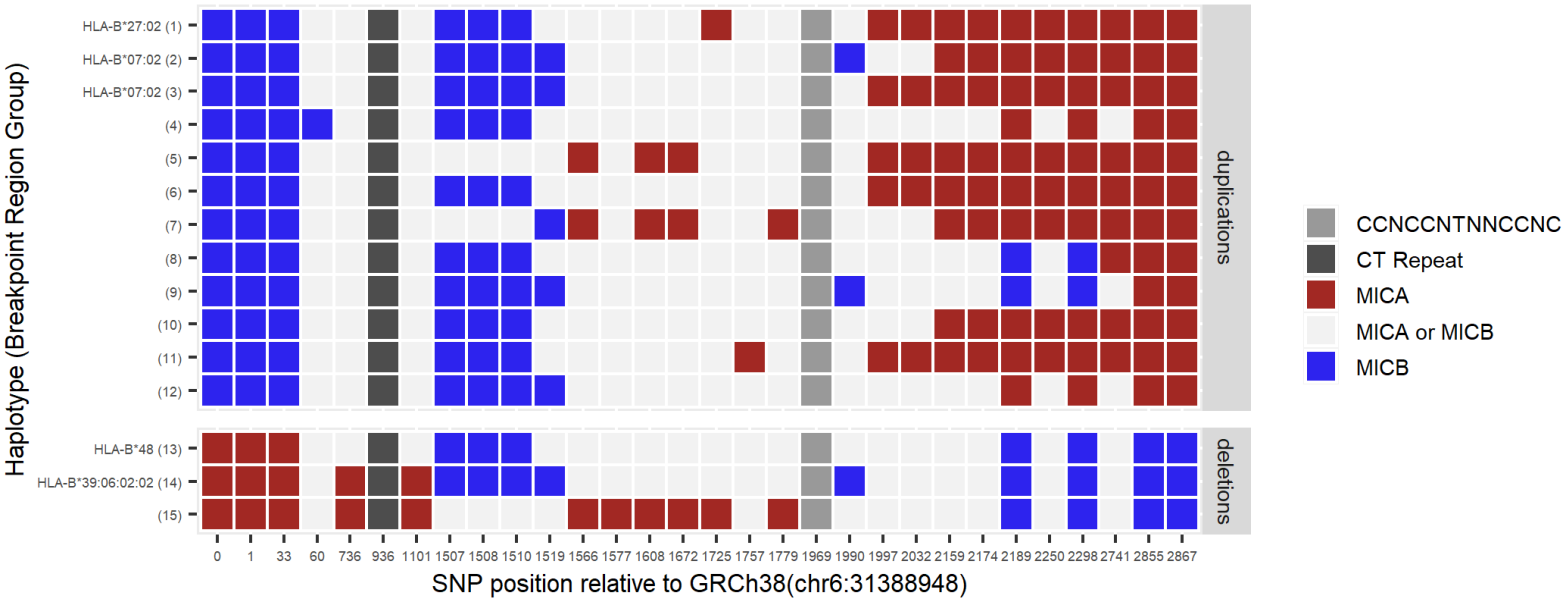
Localization of recombination sites. By aligning publicly available MHC haplotype sequences, upstream *MICA*- (red) and upstream *MICB*-specific (blue) marker SNP positions were identified in the segmental duplication regions. Other bases at these positions are shared between *MICA*- and *MICB* haplotypes (light grey). Identified marker SNPs were examined in the potential breakpoint regions of 53 *MICA* duplicated and 23 *MICA* deleted samples. Samples with the same pattern of the marker SNPs were grouped for better visibility (Breakpoint Region Group of individual samples: **Supplementary Data 3**). While Breakpoint Region Groups 1-3 and 13-14 represent common haplotypes, for groups 4-12 and 15 no haplotypes could be determined. In accordance with the proposed NAHR mechanism, *MICA* duplications are characterized by starting with *MICB*-specific bases before switching to *MICA*-specific bases. For *MICA* deletions, in full agreement with this model, it is the other way around. The switch of colors in each group narrows down the region in which the recombination has most probably happened, e.g., for the HLA-B*27:02 haplotype (Breakpoint Region Group 1) somewhere between position 1510 and 1725. Middle grey box: position of the recombination hot spot motif. Dark grey box: position of the CT dinucleotide repeat. Marker SNP positions are given relative to position chr6:31388948 of GRCh38/hg38 (**Supplementary Data 5**).

Next, we took advantage of the large variety of *MICA* duplication and -deletion haplotypes described above to characterize the underlying recombination sites. Using the duplication specific PCR and a corresponding deletion specific PCR, we amplified and sequenced the predicted recombination region of 53 *MICA* duplicated and 23 *MICA* deleted samples (**Figure 2**; **Supplementary Data 3**). Due to the very high sequence identity of the two segmental duplications, it is not possible to pinpoint the breakpoint to a particular base. Nevertheless, by aligning the sequences with available MHC haplotypes, we could narrow the recombination sites for the HLA-B*27:02:01G-linked *MICA* duplication and the HLA-B*48-linked MICA deletion haplotype down to a region of 215 and 1,474 bases, respectively (**Figure 3**, Breakpoint Region Groups 1 and 13). Interestingly, these two regions do not overlap, therefore ruling out a common ancestral event. Whenever a specific haplotype was covered by multiple samples, the sequence of the recombination region was identical, pointing to a singular recombination event underlying each haplotype (**Supplementary Data 3**, consensus_identical_group). Despite the clear variations, all studied recombination sites fall within a stretch of 2,822 bases (upstream *MICA*: chr6:31388981-chr6:31391803; upstream *MICB*: chr6:31485960-chr6:31488704) (**Figure 3**; **Supplementary Data 5**). Within this stretch, the recombination hot spot motif 5′-CCNCCNTNNCCNC-3′ can be found (upstream *MICA*: chr6:31390917, upstream *MICB*: chr6:31487832) (53, 54).

## 4 Discussion

As activating ligands of the NKG2D receptor, MIC proteins play an elaborate role in the regulation of NK and T cell function (2). This regulation is highly complex and, on the genomic level, additionally influenced by the nearly endless possible combinations of the polymorphic HLA and KIR genes (5, 38). While CNVs are common for KIR genes, germline CNVs of the classical HLA genes are rare (55). In contrast, we identified *MICA* CNVs in 1.4% of our samples. In some populations (e.g., Mexicans, South American populations), *MICA* CNV frequencies even reached 9% (56).

Despite a *MICA* duplication frequency of 1% in our samples, to our knowledge only a single case has previously been characterized (33). Since the most common haplotype, linked to HLA-B*27:02:01G, contains the frequent alleles MICA*008 and MICA*007 (42% and 5% allele frequency in the German population), copy number unaware genotyping will often result in an unsuspicious heterozygous result (35). In the future, the herein described strong linkage to HLA-B*27:02:01G may help to identify *MICA* duplications. However, it cannot substitute for copy number aware genotyping approaches, since there are HLA-B*27:02:01G haplotypes without the *MICA* duplication and several non-HLA-B*27:02:01G-linked MICA duplication haplotypes.

Our analysis of *MICA* deletions confirms the dominant HLA-B*48:01~MICB*009N associated haplotype. In Asian populations, this haplotype has previously been reported in 0.8-4% of the samples (26–28). While these numbers are supported by our data, Asian populations are not the only ones with such high frequencies (**Supplementary Data 7**). Especially in South American populations, the HLA-B*48:01~MICB*009N associated *MICA* deletion frequency is remarkably high, consistent with reports of high HLA-B*48:01 population frequencies in some indigenous populations (56–58). Individuals homozygous for this haplotype lack any functional *MICA* or *MICB* gene. In our cohort, we identified more than 50 of such homozygous individuals demonstrating that a complete absence of functional MIC proteins does not lead to major health issues, which would have prevented them from registering as potential stem cell donors (27). In addition to HLA-B*48:01:01G, we identified seven other *HLA-B* alleles with strong linkage to a *MICA* deletion that belong to the HLA-B*48, HLA-B*15, or HLA-B*39 allele groups (**Table 1**). The example of HLA-B*39:06:02G with neglectable linkage (2%) in the overall study population but strong linkage (77%) in the Mexican ethnicity suggests that more *HLA-B* linkages could be identified by higher resolution genotyping or analysis of strictly defined populations. The self-assigned populations used in this study clearly have their limitations as evident from the suspicious correlation between people self-assigned as “white” in South Africa and typical black heritage haplotypes (ZA_White, **Figure 1B**). Similarly, the ethnic group that self-assigned as “Chile Non-Indigenous” is confounded by some “Mapuche” heritage (59). Consequently, data from underrepresented and genetically diverse populations should be interpreted with caution.

For more than 1,000 *MICA* hemizygous and more than 1,200 *MICA* duplicated samples, we could not find any obvious recurrent haplotype due to the lack of physical intergenic phasing or available family data. In addition, despite our large cohort, we did not find some other previously reported MICA deletion linkages (e.g., HLA-B*07 (60), HLA-B*41 (60), HLA-B*46 (27), A*11:01~B*13:01~MICB*009N~DRB1*04:06 (28), or B*35:01~MICB*009N~DRB1*15:01 (28)). This supports our hypothesis that *MICA* deletions and -duplications are the result of frequent independent recombination events that partly may have occurred only recently.

The authors who first described the HLA-B*48:01:01G-linked *MICA* deletion in 1999 thoroughly mapped it to a 100 kB genomic deletion. They speculated that the underlying homologous recombination might have happened at a long CT dinucleotide repeat (26, 27). Our data for this haplotype support this possibility (**Figure 3**, Breakpoint Region Group 13). However, this is not the universal breakpoint for all *MICA* CNVs. Instead, our data show that breakpoints are scattered within a region of roughly 3 kb. This observation is expected for NAHR and further supported by the recombination hot spot motif 5′-CCNCCNTNNCCNC-3′ within this region, which is a binding site for the NAHR promoting zinc-finger protein PRDM9 (52–54, 61). Interestingly, allelic variations of *PRDM9* influence the recombination hot spots activity, which could be one potential reason for our observation of more *MICA* CNVs without a frequent haplotype in some populations (e.g., Chileans) (62, 63).

The significant *MICA* CNV population frequencies we report raise the question of *MICA* CNV association with disease phenotypes. So far, influence of *MICA* duplications on overall NKG2D ligand expression, and consequently NKG2D receptor activation, is unclear. For *MICA* hemizygous samples, reduced serum levels of sMICA were reported (31). Ratios of membrane-bound MICA versus sMICA levels have been shown to influence the outcome in cancer patients (8, 9). An additional gene copy of *MICA* may result in higher levels of membrane-bound MICA and may therefore be beneficial for the recognition of tumor cells by the immune system. However, the opposite could also be true and elevated *MICA* expression levels would lead to an increase in sMICA. Therefore, *MICA* CNVs should be considered in MICA/sMICA expression studies.

A *MICA* deletion, presumably in most studies the HLA-B*48-linked haplotype, has been associated with several diseases, in some instances with conflicting results, e.g., nasopharyngeal carcinoma (28, 30, 31). The most prevalent *MICA* duplication is linked to HLA-B*27:02:01G, which is one of the risk alleles for AS (64–66). An independent contribution of MICA*007:01 to AS has been discussed, but remains questionable due to its high linkage disequilibrium with HLA-B*27 (22, 24). Since our cohort of potential bone marrow donors systematically excludes disease phenotypes, the question of *MICA* CNV association with disease conditions including AS cannot be settled based on our study. In conclusion, pathogenetic relevance of *MICA* CNVs remains a fascinating possibility that warrants assessment of *MICA* genotypes in disease cohorts.

## Data availability statement

Sequence data that support the findings of this study have been deposited in GenBank with accession numbers OR060976-OR061013 (**Supplementary Data 3**). Other data are available upon reasonable request from the corresponding author.

## Ethics statement

The studies involving humans were approved by Ethics Committee of the Technische Universität Dresden, Dresden, Germany. The studies were conducted in accordance with the local legislation and institutional requirements. The participants provided their written informed consent to participate in this study.

## Author contributions

AK: Conceptualization, Formal Analysis, Investigation, Methodology, Project administration, Supervision, Visualization, Writing – original draft, Writing – review & editing. KP: Investigation, Methodology, Writing – review & editing. SK: Formal Analysis, Investigation, Methodology, Writing – review & editing. MK: Project administration, Writing – review & editing. JS: Formal Analysis, Investigation, Writing – review & editing. DS: Software, Writing – review & editing. GSchö: Formal Analysis, Investigation, Supervision, Writing – review & editing. CM: Formal Analysis, Writing – review & editing. GSchä: Formal Analysis, Writing – review & editing. AS: Resources, Supervision, Writing – review & editing. AR: Resources, Supervision, Writing – review & editing. VL: Conceptualization, Investigation, Resources, Supervision, Writing – original draft, Writing – review & editing.
