# Supplementary material for: High population frequencies of MICA copy number variations originate from independent recombination events
Source: Front Immunol. 2023 Nov 15;14:1297589. doi: 10.3389/fimmu.2023.1297589 (PMC10684724; doi:10.3389/fimmu.2023.1297589)
Supplement: Supplementary file 1 [file DataSheet_1.pdf]

Supplementary Information

High population frequencies of *MICA* copy number variations originate from independent recombination events

Sample	Exon 2		Exon 3		Exon 4/5		Gene Copies
	**Allele**	**Reads**	**Allele**	**Reads**	**Allele**	**Reads**	
**A**	A007	445	A007	175	A007	294	1
	A008	416	A008	150	A008	348	1
	**Result: *MICA*007:01 + MICA*008:01***						**2**
**B**	A007	504	A007	284	A007	358	1
	A008	481	A008	251	A008	381	1
	A011	455	A011	221	A011	303	1
	**Result: *MICA*007:01 + MICA*008:01 + MICA*011:01***						**3**
**C**	A007	182	A007	64	A007	146	1
	A008	365	A008	129	A008	395	2
	**Result: *MICA*007:01 + MICA*008:01 + MICA*008:01***						**3**

Supplementary Table 1: Identification of *MICA* duplications in neXtype: Sample A (heterozygous): *MICA* sequencing reads are equally distributed between the two alleles *MICA*007* and *MICA*008*. **Sample B** (three distinct *MICA* alleles): Even distribution of reads within each exon. **Sample C** (three copies of two distinct *MICA* alleles): number of *MICA*008* reads are about twice of *MICA*007* reads across all three exons. Exon allele groups in this table are reduced to the first field.

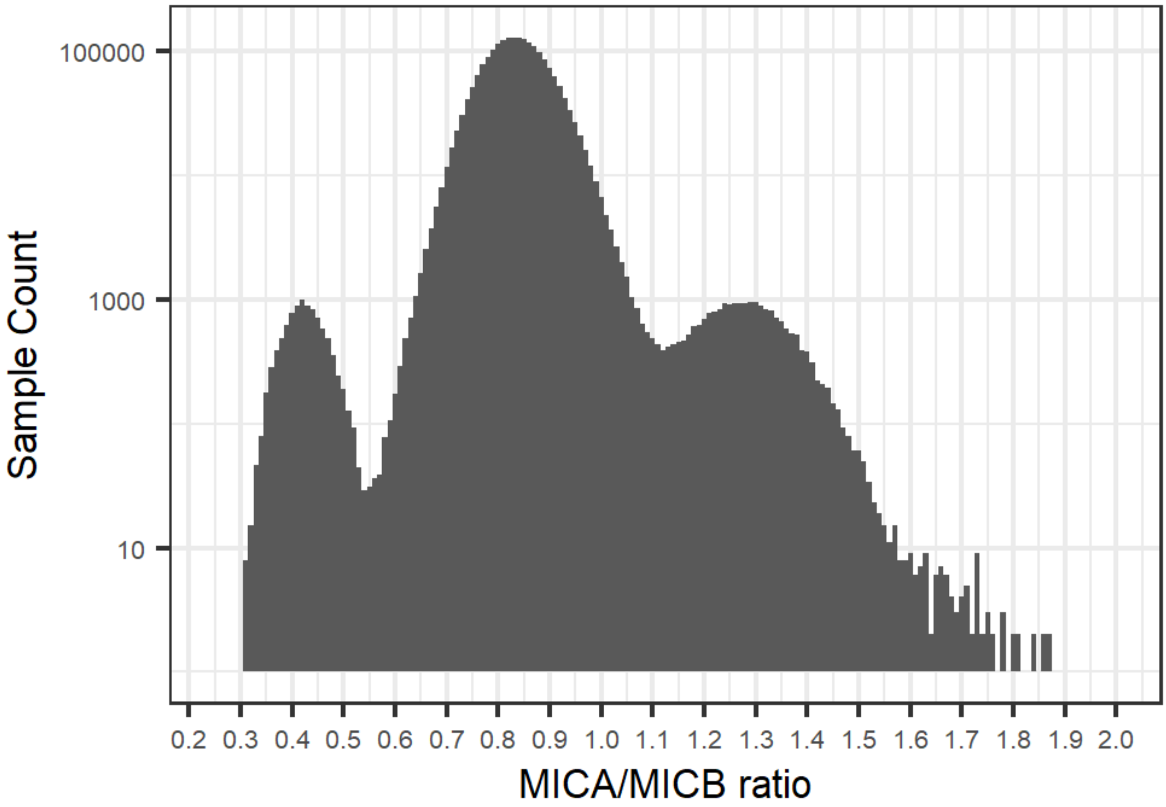

Supplementary Figure 1: Identification of *MICA* hemizygous samples. *MICA/MICB* sequencing read ratios (mean of 3 individual amplicons) for 2,089,638 million samples. The three peaks with *MICA/MICB* ratios of roughly 0.41, 0.82 and 1.27 are indicative for *MICA* gene copy numbers of 1, 2 and 3 in a sample, respectively. Samples with a *MICA/MICB* ratio <0.53 were assigned to be *MICA* hemizygous. *HLA-B*27:02:01G* homozygous samples (n=34) are characterized by *MICA/MICB* ratios >1.68 and most likely 4 copies of *MICA*. The x-axis is limited to 2.0. Not shown are 15 samples with a MICA/MICB ratio > 2.0 and the distinct haplotype *C*14:02:01G~B*51:01:01G~ MICA*009~MICA*002* (*MICA* duplicated, *MICB* deleted).
